# Exogenous laminin exhibits a unique vascular pattern in the brain via binding to dystroglycan and integrins

**DOI:** 10.1186/s12987-022-00396-y

**Published:** 2022-12-03

**Authors:** Jingsong Ruan, Karen K. McKee, Peter D. Yurchenco, Yao Yao

**Affiliations:** 1 Department of Molecular Pharmacology and Physiology, Morsani College of Medicine, University of South Florida, 12901 Bruce B. Downs Blvd., MDC 8, Tampa, FL 33612 USA; 2 Department of Pathology and Laboratory Medicine, Rutgers University-Robert W. Johnson Medical School, Piscataway, NJ USA

**Keywords:** Laminin, Dystroglycan, Integrins, Vascular pattern, Perivascular space

## Abstract

**Background:**

Unlike other proteins that exhibit a diffusion pattern after intracerebral injection, laminin displays a vascular pattern. It remains unclear if this unique vascular pattern is caused by laminin-receptor interaction or laminin self-assembly.

**Methods:**

We compared the distribution of various wild-type laminin isoforms in the brain after intracerebral injection. To determine what causes the unique vascular pattern of laminin in the brain, laminin mutants with impaired receptor-binding and/or self-assembly activities and function-blocking antibodies to laminin receptors were used. In addition, the dynamics of laminin distribution and elimination were examined at multiple time points after intracerebral injection.

**Results:**

We found that β2-containing laminins had higher affinity for the vessels compared to β1-containing laminins. In addition, laminin mutants lacking receptor-binding domains but not that lacking self-assembly capability showed substantially reduced vascular pattern. Consistent with this finding, dystroglycan (DAG1) function-blocking antibody significantly reduced the vascular pattern of wild-type laminin-111. Although failed to affect the vascular pattern when used alone, integrin-β1 function-blocking antibody further decreased the vascular pattern when combined with DAG1 antibody. EDTA, which impaired laminini-DAG1 interaction by chelating Ca^2+^, also attenuated the vascular pattern. Immunohistochemistry revealed that laminins were predominantly located in the perivascular space in capillaries and venules/veins but not arterioles/arteries. The time-course study showed that laminin mutants with impaired receptor-engaging activity were more efficiently eliminated from the brain compared to their wild-type counterparts. Concordantly, significantly higher levels of mutant laminins were detected in the cerebral-spinal fluid (CSF).

**Conclusions:**

These findings suggest that intracerebrally injected laminins are enriched in the perivascular space in a receptor (DAG1/integrin)-dependent rather than self-assembly-dependent manner and eliminated from the brain mainly via the perivascular clearance system.

**Supplementary Information:**

The online version contains supplementary material available at 10.1186/s12987-022-00396-y.

## Introduction

Laminin is a heterotrimeric protein composed of α, β, and γ chains [1, 2]. In mammals, there are five α, four β, and three γ chains, which generate many different laminin isoforms [3]. These chains form a T- or cross-shape protein with two or three short arms in the N-terminus and a long arm in the C-terminus. The short arms contain globular domains, including N-terminal (LN) domain, which are essential for laminin self-assembly [4, 5]. The long arm contains a coiled-coil domain formed by all three chains and five globular (LG) domains from α chain only, which are essential for receptor engaging [5, 6]. In the CNS, laminin is exclusively detected in the basal lamina located at the abluminal side of endothelial cells [2, 7]. Although endothelial cells, astrocytes, perivascular fibroblasts, and mural cells can all generate laminin, these cells synthesize different laminin isoforms. For example, endothelial cells predominantly produce laminin-α4β1γ1 (-411) and -511 [8–10], astrocytes predominantly make laminin-211 [9–11], and perivascular fibroblasts mainly synthesize laminin-111 [7, 12, 13]. Recent studies from our laboratory and others showed that mural cells synthesized laminin-211, -411, -511, -221, -421, and -521 [8, 14–17]. Immunohistochemistry shows that laminin-β1 is located in the basal lamina of all vascular segments in the CNS, while laminin-β2 is restricted to that of smooth muscle layer in large blood vessels [9]. These findings suggest that major laminin isoforms in the CNS under physiological conditions are laminin-111, -211, -411, and -511.

Functional studies show that laminin exerts many important functions [1, 2]. For example, loss of laminin-α1, -α5, -β1, and -γ1 leads to embryonic lethality; ablation of laminin-α2 results in muscular dystrophy and blood–brain barrier (BBB) disruption; and abrogation of laminin-β2 causes renal deficits and Pierson syndrome. In addition, conditional knockout of laminin-γ1 or -α5 in distinct cells induces BBB breakdown to different extents and affects stroke pathogenesis [15, 18–21]. Due to these important functions, laminin has been targeted in the treatment of various diseases. For example, it has been reported that laminin-111 and -521 are able to improve muscle function in Duchenne muscular dystrophy and repair glomerular basement membrane injury in Pierson syndrome, respectively [22–24]. Our preliminary study showed that laminin attenuated ischemic brain injury after intracerebral injection. In addition, laminin-integrin-β1 signaling has been shown to promote neuroblast chain formation and migration toward the injured area in stroke brains [25], indicating a beneficial role of laminin in ischemic stroke.

Many proteins (e.g. β-amyloid, ovalbumin, and low-density lipoprotein) exhibit a typical diffusion pattern, where their concentrations decrease gradually with the increase of distance from the injection site, after intracerebral injection [26, 27]. In this study, we compared the distribution of various laminin isoforms and other proteins (albumin and IgG) after intracerebral injection. Consistent with previous reports, we found that albumin and IgG displayed a typical diffusion pattern after intracerebral injection. All wild-type laminin isoforms, on the other hand, exhibited a unique vascular pattern, suggesting that they may be “concentrated/trapped” in the perivascular space in the brain. The enrichment may be caused by two possibilities: (1) exogenous laminins are incorporated into the basal lamina via self-assembly with endogenous laminins, and (2) exogenous laminins bind to their receptors, which are highly expressed at the perivascular space. We further characterized the vascular pattern of exogenous laminins in the brain, investigated the mechanisms responsible for this vascular pattern, and explored the dynamics of laminin distribution/elimination in the brain.

## Materials and methods

### Animals

C57Bl6 mice (both genders, 8–16 weeks) were maintained in the animal facility at the University of South Florida with free access to water and food. All animal procedures were approved by the Institutional Animal Care and Use Committee (IACUC) at the University of South Florida in accordance with the National Institutes of Health guidelines.

### Laminin labeling

**Fig. 1:**
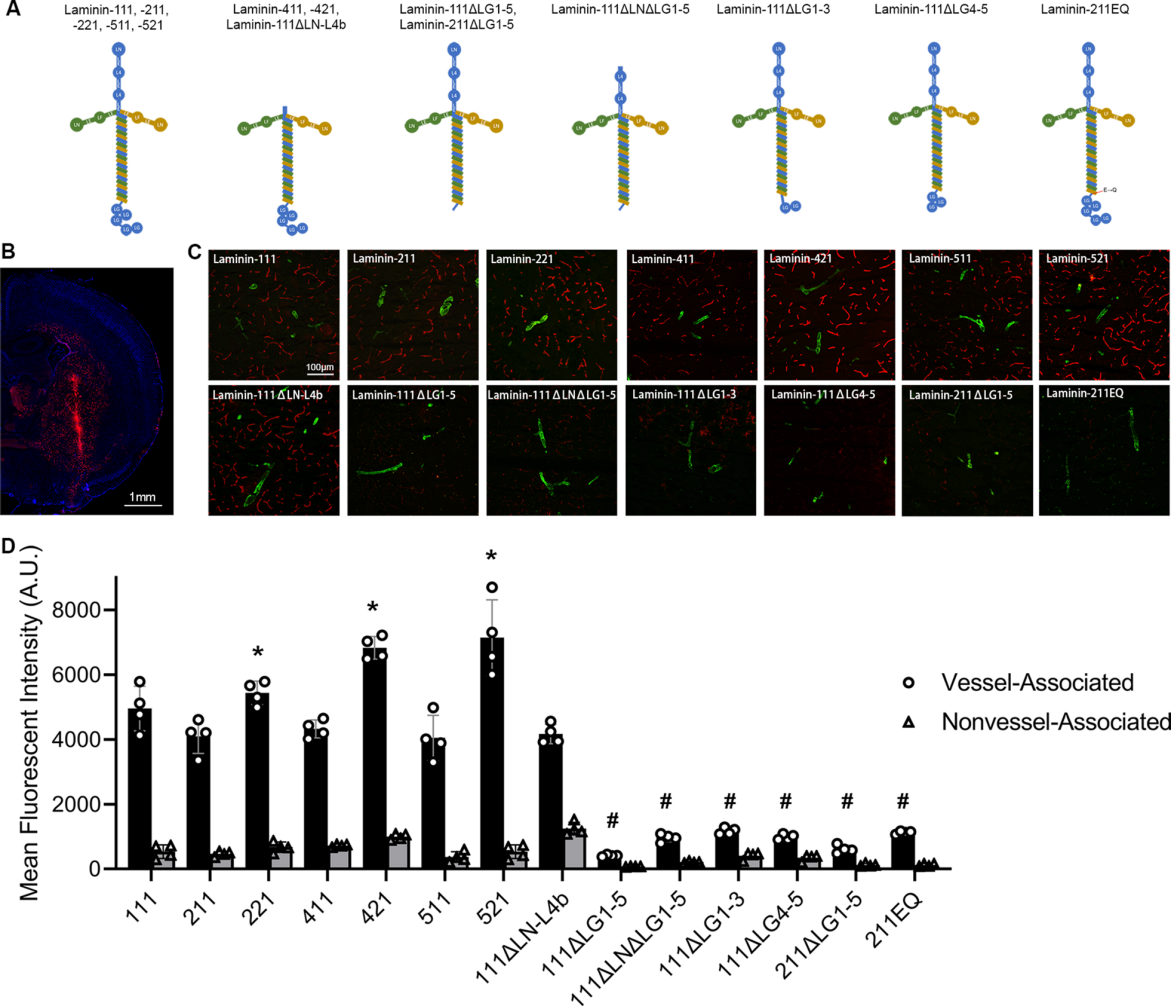
Exogenous laminins exhibit a vascular pattern in the brain via binding to their receptors rather than self-assembly. **A** Illustration of the structures of various wild-type and mutant laminins. **B** Representative low-magnification image showing the vascular pattern of intracerebrally injected laminins. **C** Representative images of Alexa-555 labeled laminins (red) and SMA (green) in the brain at 24 h after intracerebral injection. **D** Quantification of Alexa-555 fluorescent intensity in (**C**). n = 4, *p = 0.0286 compared to laminin-β1 counterparts by Mann–Whitney *U* test. ^#^*p* = 0.0286 compared to their wild-type counterparts by Mann–Whitney *U* test. Data are represented as mean ± SD. SMA, α-smooth muscle actin

Wild-type human laminin-111, -211, -221, -411, -421, -511, -521 were purchased from BioLamina. Recombinant laminin-111 proteins were composed of mouse laminin-α1 with N-terminal Myc tag, human laminin-β1 with N-terminal HA tag, and human laminin-γ1 with no tag. Recombinant laminin-211 proteins were composed of human laminin-α2 with N-terminal HA tag, human laminin-β1 with N-terminal HA tag, and human laminin-γ1 with no tag. These recombinant proteins were generated and purified as described previously [6, 28, 29]. Next, they were labeled with Alexa 555 using Microscale Protein Labeling Kit (Thermo Fisher Scientific) according to manufacturer’s instructions. All laminins were prepared in sterile PBS (137 mM NaCl, 2.7 mM KCl, 8 mM Na_2_HPO_4_, and 2 mM KH_2_PO_4_). The structures of these laminins are illustrated in Fig. 1A.

### Intracerebral injection

Mice were anesthetized with avertin (500 mg/kg body weight, i.p.) and placed on the stereotaxic instrument (RWD). A burr hole was drilled on the skull using a microdrill (Cell Point Scientific, MD, USA) and equal moles of Alexa 555-labeled laminin isoforms, Alexa 555-labeled Albumin (Invitrogen, A34786), or Alexa 555-conjugated donkey anti-rabbit IgG (Invitrogen, A31572) in 3.5 μl sterile PBS were injected into the striatum (0.5 mm posterior to bregma, 2.4 mm lateral to the midline, 3.7 mm in depth) using a 10 μl Hamilton syringe over 7 min. For some experiments, Alexa 555-labeled laminin-111 was injected into the striatum with 1.2 mM CaCl_2_ or 2.4 mM EDTA similarly as described above. For function-blocking experiments, 3.5 μl azide-free ITGB1 (BD, 555002), DAG1 (Abcam, Ab255738), or both antibodies were injected to the same coordinates 30 min before laminin injection. Hamster IgM was injected as a control. The needle was kept in the brain for 5 min after injection to prevent outflow.

### Sample collections

For CSF, mice were anesthetized and placed on the stereotaxic instrument as described above. A burr hole was drilled on the skull at the following coordinates: 0.3 mm posterior to bregma, 1 mm lateral to the midline, 2.5 mm in depth. 5 μl CSF was withdrawn from the lateral ventricle with a 10-μl syringe at 0.3 μl/minute. The 5 μl CSF was diluted in 95 μl artificial CSF, followed by Alexa 555 quantification using a fluorescent plate reader (Molecular Device, CA, USA) at 555/580 nm.

For brains, mice were anesthetized and transcardially perfused with PBS. Brains were carefully dissected out and immediately frozen in dry ice, followed by sectioning with a cryostat (CryoStar NX50, Thermo Scientific, USA). Coronal serial sections covering 1 mm brain tissue (500 μm anterior and 500 μm posterior to the injection site) were collected.

### Immunofluorescence and image analyses

Laminin distribution in the brain was assessed using fluorescent and confocal microscopes. Arteries and arterioles were marked by α-smooth muscle actin (SMA) antibody (F3777, Sigma). Laminin levels were quantified using ImageJ. For total laminin quantification, mean fluorescent intensity of Alexa 555 signal defined as the integrated fluorescence intensity normalized to total area of the image was used. For vessel-associated laminin quantification, Alexa 555 signal that has a vascular pattern was manually selected in each image. The integrated fluorescence intensity of selected regions was normalized to total area of selected regions to determine the mean fluorescence intensity. Similarly, the mean fluorescence intensity of non-vessel-associated laminin was quantified as the integrated fluorescence intensity of non-vessel-associated Alexa 555 signal normalized to non-vessel area. The non-vessel-associated Alexa 555 signal was calculated by subtracting vessel-associated Alexa 555 signal from total Alexa 555 signal. Three images per section and five serial sections evenly distributed across the injection site (identified by needle track) were used for quantification.

Laminin contact was defined as the percentage of Alexa 555-positive vessel length over total CD31-positive vessel length. Laminin coverage was defined as the percentage of Alexa 555-positive vessel area over total CD31-positive vessel area. Vessel length and vessel area were determined using both laminin (Alexa 555) and CD31 signals using AngioTool. Three images per section and five serial sections evenly distributed across the injection site (identified by needle track) were used for quantification.

### Statistical analysis

Experimental data were analyzed by GraphPad Prism 9 software (GraphPad Software, San Diego, CA, USA). All values were expressed as Mean ± SD. Mann–Whitney *U* test was used to determine significance between experimental groups. Sample number (n) represents biological replicates. A p-value < 0.05 was deemed to be statistically significant.

## Results

### Laminins demonstrate a unique vascular pattern after intracerebral injection

To characterize the distribution pattern of various laminin isoforms, Alexa 555-labeled laminin-α1β1γ1 (-111), -211, -221, -411, -421, -511, and -521 (Fig. 1A) were intracerebrally injected into wild-type mice. Albumin, IgG, and heat-inactivated laminin isoforms were injected as controls. Like most proteins, albumin (Additional file 1: Fig. S1A) and IgG (Additional file 1: Fig. S1B) displayed a typical diffusion pattern. Interestingly, although all heat-inactivated laminin isoforms exhibited a diffusion pattern (Additional file 1: Fig. S1C, D), their wild-type (non-inactivated) counterparts showed a vascular pattern 24 h after injection (Fig. 1B, C), suggesting that the tertiary structure of laminin is essential for the vascular pattern.

### β2-containing laminins have a stronger vascular pattern than β1-containing laminins

Although a vascular pattern was observed in all laminin isoforms, different laminin isoforms showed distinct fluorescent intensity. Specifically, all β1-containing laminins (laminin-111, -211, -411, and -511) displayed comparable fluorescent intensity, while β2-containing laminins (laminin-221, -421 and -521) exhibited much stronger fluorescence intensity compared to their β1-containing counterparts (Fig. 1C, D). These findings suggest that laminin-α chains have similar affinity for the basal lamina, while laminin-β2 chain may have a higher affinity for the basal lamina than laminin-β1 chain.

### Laminin-receptor interaction rather than self-assembly mediates the vascular pattern

To determine whether the vascular pattern is mediated by laminin self-assembly or laminin-receptor interaction, we generated laminin-111 mutant unable to self-assemble (lacking α1 LN-L4b domains, termed laminin-111ΔLN-L4b), laminin-111 and -211 mutants unable to bind their receptors (lacking LG1-5 domains in α1 and α2 chains, termed laminin-111ΔLG1-5 and -211ΔLG1-5), and laminin-111 mutant unable to self-assemble and bind their receptors (lacking both LN and LG1-5 domains in α1 chain, termed laminin-111ΔLNΔLG1-5) (Fig. 1A). Laminin-111ΔLN-L4b demonstrated comparable distribution pattern and fluorescent intensity as wild-type laminin-111 at 24 h after intracerebral injection (Fig. 1C, D), indicating a minimal role of laminin-α1 LN-L4b domains and thus self-assembly in the vascular pattern. Although laminin-111ΔLG1-5, -211ΔLG1-5, and -111ΔLNΔLG1-5 also showed a vascular pattern, their fluorescent intensity was significantly reduced (Fig. 1C, D), highlighting an important role of laminin LG domains and thus receptor-binding in the vascular pattern.

**Fig. 2:**
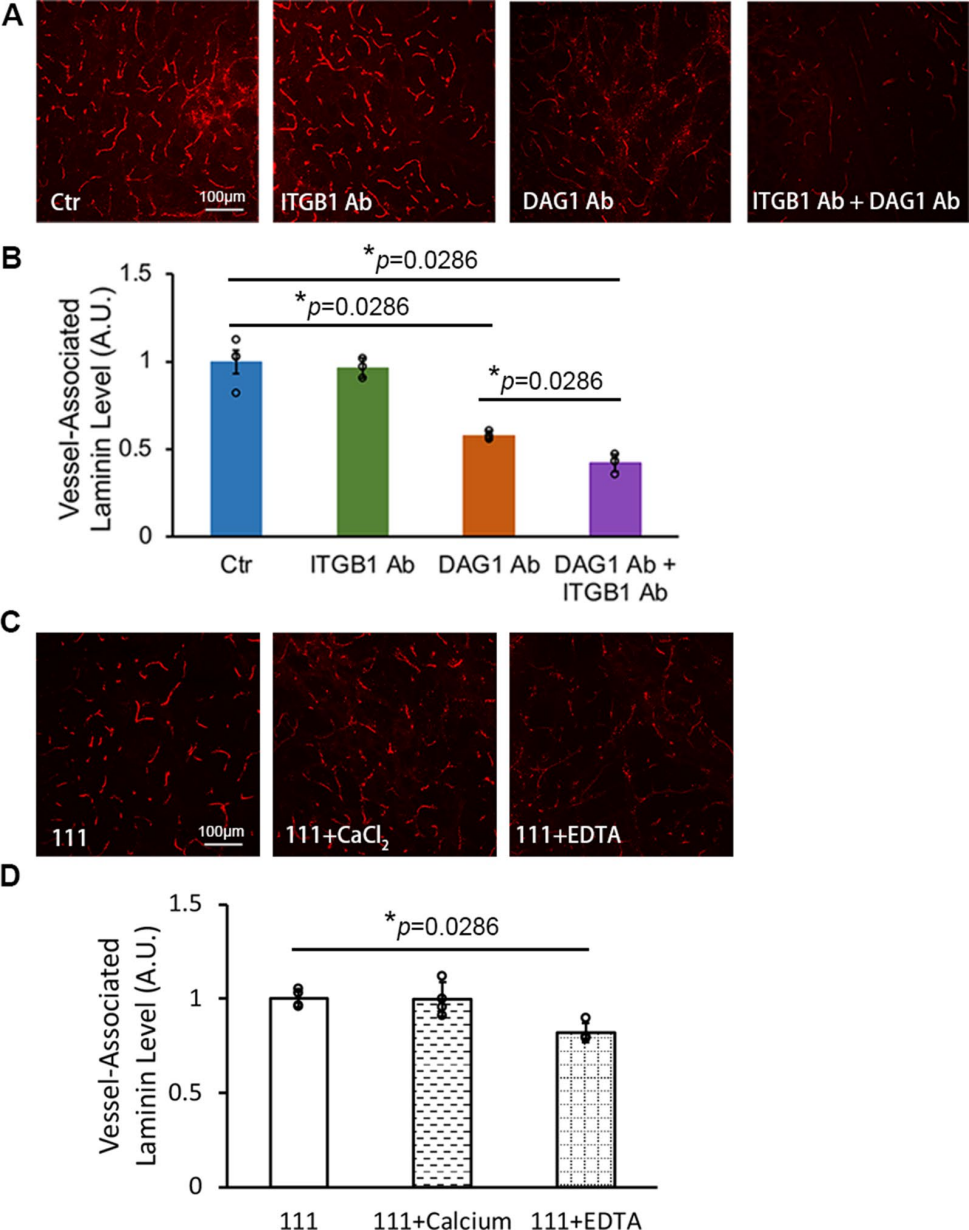
Laminin receptors mediate the vascular pattern of intracerebrally injected laminins. **A** Representative images of Alexa-555 labeled laminin-111 (red) in the brain at 24 h after intracerebral injection in the presence of IgM control, ITGB1 function-blocking antibody, DAG1 function-blocking antibody, and both ITGB1 and DAG1 function-blocking antibodies. **B** Quantification of Alexa-555 fluorescent intensity in blood vessels in **A**. n = 4, *p = 0.0286 by Mann–Whitney *U* test. **C** Representative images of Alexa-555 labeled laminin-111 (red) in the brain at 24 h after intracerebral injection in the presence of CaCl_2_ or EDTA. **D** Quantification of Alexa-555 fluorescent intensity in blood vessels in **C**. n = 4, *p = 0.0286 by Mann–Whitney U test. Data are represented as mean ± SD. DAG1, dystroglycan; ITGB1, integrin-β1; EDTA, ethylenediaminetetraacetic acid

To identify which receptor(s) mediates the vascular pattern, we blocked laminin receptors with function-blocking antibodies. Integrins and DAG1 are two major laminin receptors in the CNS [2, 30, 31]. Blocking integrin-β1 (ITGB1), a common subunit for most classical laminin receptors [31], failed to affect the vascular pattern or fluorescent intensity of laminin-111 when used alone (Fig. 2A, B). DAG1 blocking antibody, on the other hand, significantly reduced the level of vessel-associated laminin-111 (Fig. 2A, B). Interestingly, the fluorescent intensity of vessel-associated laminin-111 was further diminished in the presence of both DAG1 and ITGB1 blocking antibodies (Fig. 2A, B). These results suggest that both DAG1 and ITGB1 mediate the vascular pattern with the former having a possibly more important role. Since laminin-DAG1 binding is strictly calcium-dependent [32], we further investigated the distribution pattern of laminin-111 in the presence of calcium chloride or EDTA. Calcium chloride failed to affect the fluorescent intensity of vessel-associated laminin-111 at 24 h after intracerebral injection (Fig. 2C, D), possibly due to endogenous calcium in the extracellular space in the brain. In contrast, EDTA reduced the fluorescent intensity of vessel-associated laminin-111 at 24 h after intracerebral injection (Fig. 2C, D). Consistent with these findings, laminin-111 mutant with impaired DAG1-binding activity (lacking α1 LG4-5 domains, termed laminin-111ΔLG4-5) exhibited substantially decreased fluorescent intensity in the vasculature (Fig. 1C, D), again indicating an important role of DAG1 in the formation of vascular pattern. Interestingly, laminin-111 mutant lacking α1 LG1-3 domains (termed laminin-111ΔLG1-3) and laminin-211 mutant containing a Glu to Gln mutation in the C-terminal tail of γ1 chain (termed laminin-211EQ), both of which are defective in interacting with classical laminin-binding integrins [33, 34], displayed significantly reduced fluorescent intensity in the blood vessels compared to their respective wild-type counterparts (Fig. 1C, D), highlighting an essential role of integrins in vascular pattern formation. Given the minimal role of ITGB1 blocking alone in vascular pattern formation (Fig. 2A, B), we speculate that other laminin-binding integrins, such as integrin-α6β4, may mediate the vascular pattern of intracerebrally injected laminins. Together, these results suggest that exogenous laminins form a vascular pattern in the brain predominantly via interacting with their receptors highly expressed at the cerebral vasculature rather than self-assembly with endogenous laminins in the basal lamina.

### Characterization of the vascular pattern of exogenous laminins in the brain

**Fig. 3:**
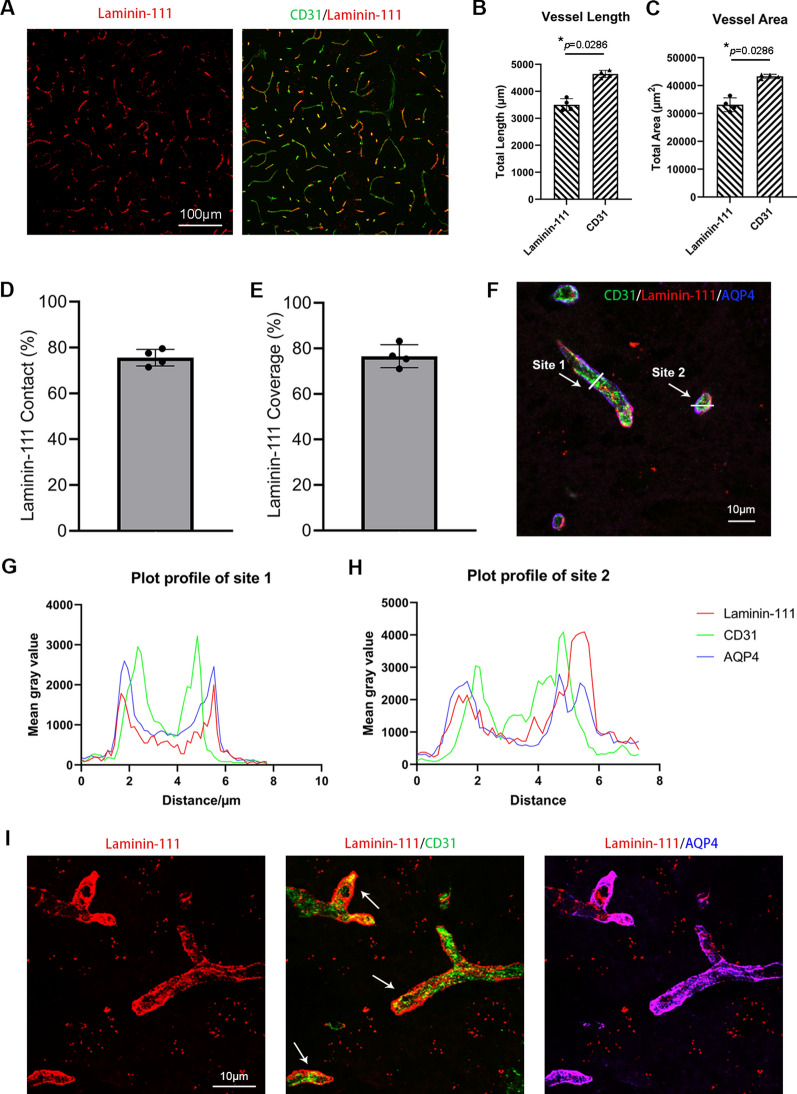
Exogenous laminin-111 is enriched in the perivascular space in cerebral vasculature. **A** Representative low-magnification images of Alexa-555 labeled laminin-111 (red) and CD31 (green) in the brain at 24 h after intracerebral injection. **B** Quantification of vessel length calculated with laminin-111 and CD31 signals. n = 4, *p = 0.0286 by Mann–Whitney U test. **C** Quantification of vessel area calculated with laminin-111 and CD31 signals. n = 4, *p = 0.0286 by Mann–Whitney *U* test. **D** Quantification of laminin-111 contact. n = 4. **E** Quantification of laminin-111 coverage. n = 4. **F** Representative high-magnification image of CD31 (green), Alexa-555 labeled laminin-111 (red), and AQP4 (blue) in the brain at 24 h after intracerebral injection. White arrows indicate two sites, where spatial profiles of fluorescence intensity were performed. **G**, **H** Spatial profiles of CD31 (green), laminin-111 (red), and AQP4 (blue) along white lines crossing representative capillaries in site 1 (**G**) and site 2 (**H**) in **F**. **I** Representative high-magnification images of CD31 (green), Alexa-555 labeled laminin-111 (red), and AQP4 (blue) showing the distribution of laminin-111 along the longitudinal axis of blood vessels. White arrows indicate the vascular pattern of laminin-111 at the abluminal side of endothelial cells. Data are represented as mean ± SD

Next, we characterized the vascular pattern of exogenous laminins in the brain using Alexa-555 labeled laminin-111 and -211. Immunohistochemical analysis showed that all laminin-111 signal merged with endothelial marker CD31, while not all CD31 signal merged with laminin-111 at 24 h after intracerebral injection (Fig. 3A). Consistent with this observation, vessel length (Fig. 3B) and vessel area (Fig. 3C) calculated using laminin-111 were significantly lower than that calculated using CD31. Quantification revealed substantially reduced laminin-111 contact (Fig. 3D) and laminin-111 coverage (Fig. 3E). Similar results were observed for laminin-211 (Additional file 1: Fig. S2A–E). These results suggest that exogenous laminins are distributed to some but not all vessels in the brain. Further analysis showed that all intracerebrally injected laminin isoforms were predominantly found in SMA-negative blood vessels (capillaries and venules/veins) but not SMA-positive arterioles/arteries (Fig. 1C), which is consistent with the perivascular route of waste clearance in the brain [35, 36]. Together, these findings suggest that exogenous laminins may be cleared from the brain, at least partially, via the perivascular system.

### Exogenous laminins are eliminated from the brain through the perivascular system

To study the dynamics of laminin distribution and elimination in the brain, we examined the location of injected laminin-111 and -211 by immunohistochemistry. Laminin-111 co-localized with endothelial marker CD31 and astrocyte-endfoot marker AQP4 (Fig. 3F). Interestingly, the peak of laminin-111 signal was found in the abluminal side of CD31 and co-localized with AQP4 (Fig. 3G-I). Similar finding was observed for laminin-211 at 24 h after intracerebral injection (Additional file 1: Fig. S2F–H). These results suggest that exogenous laminins are sandwiched between endothelial cells and astrocytes (in the perivascular space). In addition, we failed to detect laminin-111 or -111ΔLG1-5 in Iba-1^+^ microglia/macrophages at 24 h after intracerebral injection (Additional file 1: Fig. S3A), indicating a minimal role of microglia/macrophage-mediated phagocytosis in exogenous laminin elimination. Similarly, no laminin-111 or -111ΔLG1-5 was detected in the blood at various time points after intracerebral injection (Additional file 1: Fig. S3B), suggesting that exogenous laminins are unlikely transported across the BBB and eliminated via systemic circulation. Given that the perivascular space is a major component of the perivascular route of waste clearance, these findings together suggest that exogenous laminins are mainly eliminated from the brain via the perivascular system.

**Fig. 4:**
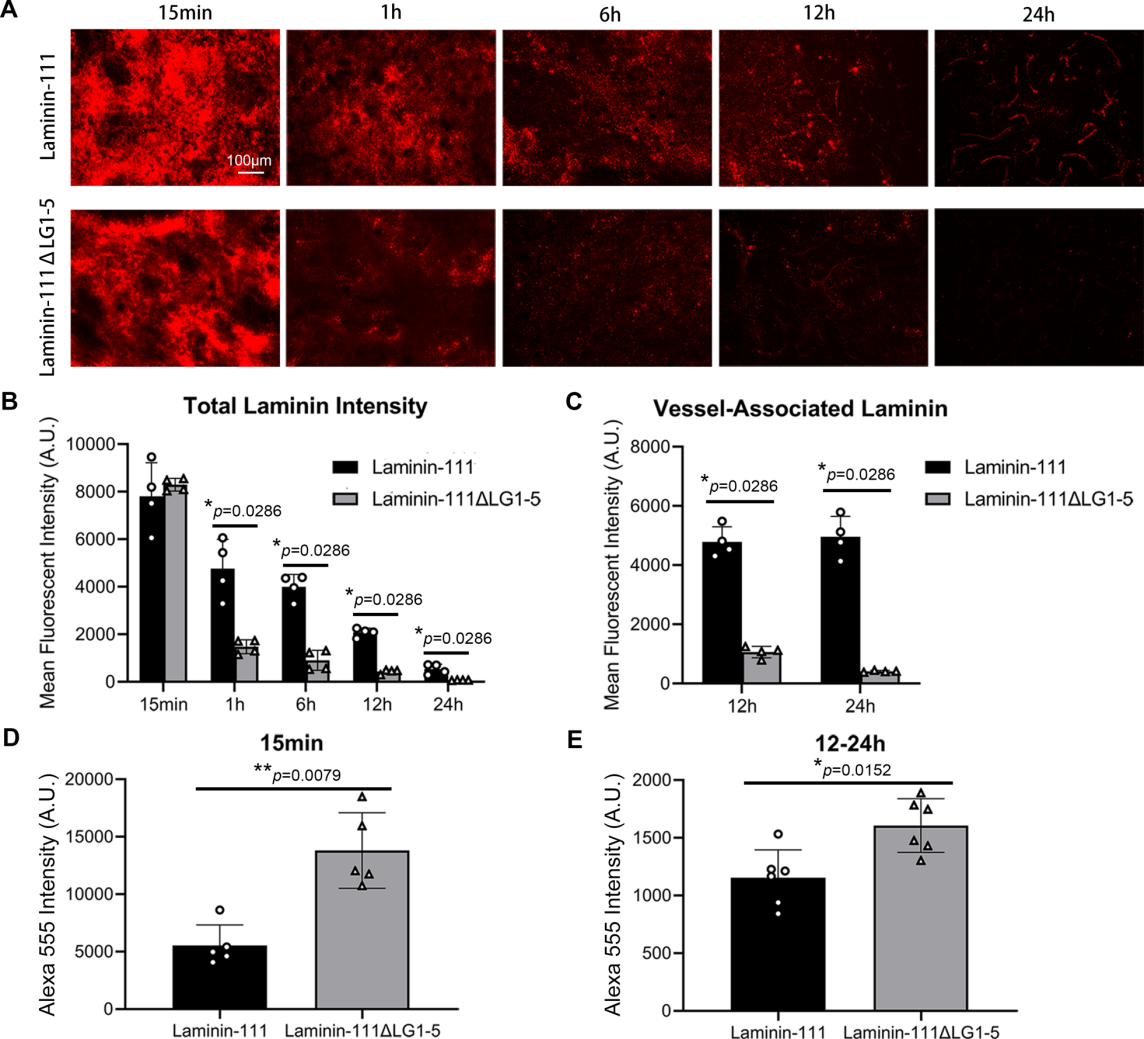
Exogenous laminin-111 and -111ΔLG1-5 are eliminated from the brain via the perivascular system. **A** Representative images of Alexa-555 labeled laminin-111 (red) and -111ΔLG1-5 (red) in the brain at various time points after intracerebral injection. **B** Quantification of total laminin levels at each time point in **A**. n = 4, *p = 0.0286 by Mann–Whitney U test. **C** Quantification of vessel-associated laminin levels at 12 and 24 h after intracerebral injection in **A**. n = 4, *p = 0.0286 by Mann–Whitney U test. **D**, **E** Quantification of Alexa-555 fluorescent intensity in the CSF at 15 min (**D**) and 24 h (**E**) after intracerebral injection. n = 5–6, *p = 0.0152 and **p = 0.0079 by Mann–Whitney U test. Data are represented as mean ± SD

To further test this hypothesis, we examined the distribution pattern and fluorescent intensity of laminin-111/111ΔLG1-5 and laminin-211/211ΔLG1-5 at various time points after intracerebral injection. Both laminin-111 and -111ΔLG1-5 exhibited a diffusion pattern up to 6 h after intracerebral injection and the vascular pattern was observed at 12 and 24 h after intracerebral injection (Fig. 4A). Quantification showed that the levels of laminin-111 and -111ΔLG1-5 decreased gradually over time (Fig. 4B). Although both laminin isoforms exhibited comparable fluorescent intensity at 15 min after intracerebral injection, laminin-111ΔLG1-5 showed more dramatic reduction at later time points (Fig. 4B). Laminin-211 and -211ΔLG1-5 exhibited similar dynamic changes as laminin-111 and -111ΔLG1-5 after intracerebral injection (Additional file 1: Fig. S4A, B). Since vascular pattern was observed at 12 and 24 h after intracerebral injection, we further quantified the levels of vessel-associated laminins at these two time points. Compared to their respective wild-type counterparts, laminin-111ΔLG1-5 (Fig. 4C) and -211ΔLG1-5 (Additional file 1: Fig. S4C) displayed substantially lower levels at both 12 and 24 h after intracerebral injection. These results suggest that both wild-type and mutant laminins are cleared from the brain via the perivascular system with the mutant ones having a higher clearance rate. The enhanced elimination of mutant laminins is probably caused by their lack of receptor-binding capability, which diminishes their retention in the perivascular space. Next, we further examined laminin levels in the CSF, another major component of the perivascular system [35, 36]. Compared to their wild-type counterparts, both laminin-111ΔLG1-5 (Fig. 4D) and -211ΔLG1-5 (Additional file 1: Fig. S4D) exhibited significantly higher levels in the CSF at 15 min after intracerebral injection, again indicating faster elimination from the brain. Interestingly, the CSF level of laminin-111ΔLG1-5 remained substantially higher than that of laminin-111 at 12–24 h after intracerebral injection (Fig. 4E). Together, these results suggest that exogenous laminins enter the perivascular space of SMA^−^ blood vessels (capillaries and venules/veins) and are eliminated from the brain via the perivascular system.

## Discussion

In this study, we examined the distribution of exogenous laminin isoforms in brain parenchyma. Although all laminin isoforms displayed a vascular pattern 24 h after intracerebral injection, β2-containing laminins showed significantly higher levels in blood vessels compared to β1-containing laminins, indicating stronger binding between β2-containing laminins and laminin receptors in the perivascular space. Consistent with this finding, laminin-421 exhibits higher binding activity to CD146, a vascular receptor for α4-containing laminins [37], compared to laminin-411 [38]. Similarly, it has been reported that laminin-β2 has a higher binding affinity for integrin α subunits that contain the X2-type region (e.g. integrin-α3β1) compared to laminin-β1 [39]. Subsequent research has revealed that the C-terminal 20 amino-acid residues in the coiled-coil domain are responsible for the enhanced integrin binding by β2-laminins [39].

What is the functional significance of different receptor-binding affinity between laminin-β1 and -β2? It is speculated that the difference in receptor-binding affinity may differentially regulate laminin expression and/or function. For example, it has been shown that laminin-β2 is gradually replaced by laminin-β1 during kidney and cartilage development [40, 41], whereas laminin-β1 seems to be replaced by laminin-β2 during skeletal muscle development [42]. Similarly, laminin-β1 is mainly found in arterial SMCs in early fetal stage, while laminin-β2 is predominantly expressed in these cells in adulthood [43]. Like during development, laminin β switch is also found in aging or injury conditions. It has been reported that laminin-β1 is up-regulated, while laminin-β2 is down-regulated in endothelial cells from aged mice or after acute injury [44]. This β2-to-β1 switch has been shown to change the functional properties and phenotypes of endothelial cells [44]. In addition, it has been reported that β2-containing laminins support stronger cell adhesion in an integrin-dependent manner [39, 45]. In the CNS, most laminin isoforms within the basal lamina are of the β1 type and laminin-β2 expression is restricted to the smooth muscle layer in adulthood [2, 7, 9, 10, 13]. This expression pattern suggests that laminin-β1 is likely sufficient to maintain normal functions in the CNS under homeostatic conditions, whereas laminin-β2, which has higher affinity for laminin receptors and more strongly activates integrin receptors, is needed to repair injury in pathological conditions.

The receptor-binding regions of laminin have been mapped to the LG1-5 domains in the α subunit and the Glu residue in the C-terminus of γ1 subunit [33, 46, 47]. It remains unclear how exactly laminin β subunit regulates laminin-receptor interaction. It is speculated that laminin β subunits may directly interact with laminin receptors and/or indirectly regulate laminin-receptor binding affinity by fine-tuning the conformation of receptor-binding sites. These possibilities need to be examined in future studies.

Using various laminin mutants, we further showed that loss of LG1-5 but not LN-L4b domains dramatically reduced the fluorescent intensity of vessel-associated laminins in the brain. Given that the LN-L4b domain is essential for laminin self-assembly [4, 5] and the LG1-5 domains are required for receptor binding [5, 6], these findings suggest that the vascular pattern is mediated predominantly by laminin-receptor interaction rather than self-assembly. Using function-blocking antibodies and laminin mutants unable to interact with DAG1 (laminin-111ΔLG4-5) or integrins (laminin-111ΔLG1-3 and laminin-211EQ), we further reported that both DAG1 and integrins mediated the vascular pattern with DAG1 having a possibly more important role. It should be noted, however, that weak vascular pattern was still observed when both DAG1 and ITGB1 were blocked or when laminin mutants with impaired DAG1/integrin-binding activity were used. This may be caused by the contribution of other laminin receptors, such as galactosyl-sulfatides.

Dystroglycanopathies are a group of diseases characterized by impaired laminin-DAG1 interaction, including Walker-Warburg syndrome [48], muscle-eye-brain disease [49], and Fukuyama congenital muscular dystrophy [50]. Patients with these disorders usually develop muscle dystrophy, brain deformity, and BBB disruption. For example, *POMT1* (protein O-mannosyltransferase) mutation disrupts the glycosylation of laminin binding site on DAG1, leading to Walker-Warburg syndrome—a severe neuronal migration disorder, in which patients exhibit severe muscular dystrophy, brain malformations, abnormal eye development, and BBB impairment [51, 52]. *POMGNT1* (protein O-linked-mannose beta-1,2-N-acetylglucosaminyltransferase 1) mutation impairs DAG1 glycosylation and thus DAG1-ligand interaction, leading to muscle-eye-brain disease, which is characterized by muscle weakness, eye development defects, and BBB disruption [52, 53]. Similarly, *fukutin* mutation compromises the glycosylation of DAG1, leading to Fukuyama congenital muscular dystrophy, a milder dystroglycanopathy characterized by progressive muscle weakness [54, 55] and brain vessel dysfunction [56]. In addition, mutations in laminin and DAG1 also cause dystroglycanopathies. It has been reported that laminin-α2 null mice, a mouse model of congenital muscular dystrophy, develop BBB impairment [57]; loss of astrocytic laminin leads to severe BBB disruption and spontaneous intracerebral hemorrhage [58]; and ablation of laminin-γ1 in PDGFRβ^+^ cells leads to muscular dystrophy and BBB breakdown in the C57Bl6/FVB mixed background [16, 59]. Similarly, a homozygous c.743C > del frameshift mutation in DAG1 causes complete loss of DAG1, leading to brain deformity, hydrocephalus, subependymal hemorrhages [60]. Together, these findings suggest that laminin-DAG1 interaction is essential for BBB integrity maintenance.

How is intracerebrally injected laminin eliminated from the brain? One possible mechanism is via the perivascular route of waste clearance, in which CSF flushes the brain and removes metabolic wastes via the perivascular space [35, 61]. There is evidence showing that this perivascular system contributes to the elimination of multiple macromolecules in the CNS. For example, it has been shown that the efflux and drainage of β-amyloid, ovalbumin, and low-density lipoprotein are impaired in mice with ablated meningeal lymphatic vessels [27]. Similarly, ovalbumin clearance is compromised in K14-VEGFR3-Ig transgenic mice, which have no dura matter lymphatic vasculature [26]. In addition, intracerebrally injected β-amyloid and mannitol are eliminated from the brain through the perivascular pathway [62]. Furthermore, it has been suggested that impaired perivascular clearance pathway exacerbates or even induces pathogenic accumulation of tau [63].

In this study, we showed that intracerebrally injected laminins were detected in the abluminal side of endothelial cells and co-localized with astrocyte endfeet, supporting a perivascular localization. In addition, exogenous laminin was found exclusively in SMA^−^ blood vessels (capillaries and venules/veins) but not SMA^+^ arterioles/arteries, a pattern consistent with the perivascular route of waste clearance [35, 36]. Furthermore, a time-course study revealed that exogenous laminins displayed a diffusion pattern early after injection, which changed to a vascular pattern at later time points. The switch from diffusion pattern to vascular pattern suggests that exogenous laminins may be cleared from the brain via the perivascular system. Compared to laminin-111 and -211, laminin-111ΔLG1-5 and -211ΔLG1-5 exhibited a faster elimination rate, which could be explained by reduced retention in the perivascular space due to their lack of receptor-binding capability. Echoed with this finding, significantly higher levels of laminin-111ΔLG1-5 and -211ΔLG1-5 were detected in the CSF after intracerebral injection compared to their wild-type counterparts. Together, these results support that exogenous laminin is eliminated from the brain via the perivascular system. It should be noted that the mechanisms by which interstitial waste enters perivascular space remain largely understudied. It has been reported that astrocytic endfeet form a barrier between interstitial space and perivascular space [64]. Given its large molecular weight, it is unlikely that laminin crosses astrocytic endfeet through passive diffusion. Based on the expression of various laminin receptors in astrocytic endfeet, it is speculated that laminin may be transported through endocytosis. Another possibility is that laminin may enter the perivascular space by crossing the gaps between astrocytic endfeet.

In addition to the perivascular route of waste clearance, laminin may also be transported across the BBB and eliminated via systemic circulation, which is responsible for the clearance of more than 70% of extracellular Aβ [65]. However, we failed to detect laminins in the blood up to 24 h after intracerebral injection, indicating a minimal contribution of systemic circulation in laminin elimination. It should be noted that we cannot exclude the possibility that this negative result is due to the low levels of laminins injected. Similarly, laminin may also be phagocytosed/taken up by microglia/macrophages or degraded locally by endogenous proteases. The lack of co-localization between Alexa 555 (laminins) and Iba-1 (microglia/macrophages) in the brain at 24 h after intracerebral injection suggests that microglia/macrophage-mediated phagocytosis is unlikely the major route for laminin elimination from the brain. However, we cannot exclude the possibility that intracerebrally injected laminins are degraded locally by proteases.

Our findings have important implications in the fields of biomedical research and targeted drug delivery. First, intracerebral injection of fluorescently labeled laminins allows labeling of capillaries and venules/veins but not arterioles/arteries. This is useful when different types (arteries/arterioles vs. capillaries and venules/veins) of cerebral blood vessels need to be distinguished in live mice (e.g. during two-photon imaging). Next, the unique vascular pattern of exogenous laminin may be utilized to target cerebral blood vessels and/or pericytes. For example, laminin LG domains may be conjugated to β-amyloid neutralizing antibody to enhance their concentrations in cerebral blood vessels in the treatment of cerebral amyloid angiopathy. Given that pericyte defects are observed in stroke [59], therapeutics reversing these defects may be conjugated to laminin LG domains to increase their delivery to pericytes. One potential issue with this approach is that laminin LG domains may compete with and displace endogenous laminin in the basal lamina. This may alter the composition/balance of different laminin isoforms in the basal lamina, leading to undesired consequences. However, we think this is unlikely for the following four reasons. First, laminin is integrated into the basal lamina at high degree by interacting with multiple molecules. It would be difficult for exogenous laminin to break the interactions already formed and displace endogenous laminin. Second, we failed to detect exogenous laminin (Alexa-555) in the brain at 48 h after intracerebral injection (not shown). If exogenous laminin efficiently displaces endogenous one and gets incorporated into the basal lamina, we should be able to detect it, since laminin in the basal lamina has low turnover rate [1, 2, 7, 66, 67]. Third, no differences in the distribution patterns of self-polymerization competent and incompetent laminins were detected (Fig. 1C, D). Fourth, no obvious abnormalities have been observed in mice with intracerebral injection of various laminin isoforms, including laminin-111 and -521, in our laboratory. In addition, no undesired CNS effects were reported in mice with intraperitoneal [68] or intramuscular [69] injection of laminin-111. Therefore, we expect potential toxicity of this approach to be minimal.

## Conclusions

In summary, we demonstrate that laminins exhibit a unique vascular pattern after intracerebral injection with β2-containing laminins having a higher affinity for the vessels compared to β1-containing ones, and that this vascular pattern is mediated by laminin-DAG1/integrin interaction rather than self-assembly. In addition, we also show that laminin is distributed to the perivascular space in capillaries and venules/veins but not arterioles/arteries after intracerebral injection, and eliminated from the brain via the perivascular clearance system. These findings may have important implications in biomedical research and targeted drug delivery.

## Supplementary Information

**Additional file 1: Figure S1.** Albumin, IgG, and heat-inactivated laminins display a typical diffusion pattern at 24 h after intracerebral injection. **Figure S2.** Exogenous laminin-211 is enriched in the perivascular space in cerebral vasculature. **Figure S3.** Exogenous laminin-111/laminin-111ΔLG1-5 are not eliminated from the brain by microglia/macrophage-mediated phagocytosis or systemic circulation. **Figure S4.** Exogenous laminin-211/laminin-211ΔLG1-5 are eliminated from the brain via the perivascular system.

## Data Availability

The data used and/or analyzed during the current study are available from the corresponding author on reasonable request.

## Abbreviations

BBB: Blood–brain barrier
CSF: Cerebral-spinal fluid
DAG1: Dystroglycan
LG: Laminin globular domains
LN: Laminin N-terminal domain
SMA: α-Smooth muscle actin
