# Supplementary material for: Exogenous laminin exhibits a unique vascular pattern in the brain via binding to dystroglycan and integrins
Source: Fluids Barriers CNS. 2022 Dec 3;19:97. doi: 10.1186/s12987-022-00396-y (PMC9719645; doi:10.1186/s12987-022-00396-y)
Supplement: Supplementary file 1 — Additional file 1: Figure S1. Albumin, IgG, and heat-inactivated laminins display a typical diffusion pattern at 24 h after intracerebral injection. Figure S2. Exogenous laminin-211 is enriched in the perivascular space in cerebral vasculature. Figure S3. Exogenous laminin-111/laminin-111ΔLG1-5 are not eliminated from the brain by microglia/macrophage-mediated phagocytosis or systemic circulation. Figure S4. Exogenous laminin-211/laminin-211ΔLG1-5 are eliminated from the brain via the perivascular system. [file 12987_2022_396_MOESM1_ESM.docx]

**Supplementary Figure**

**
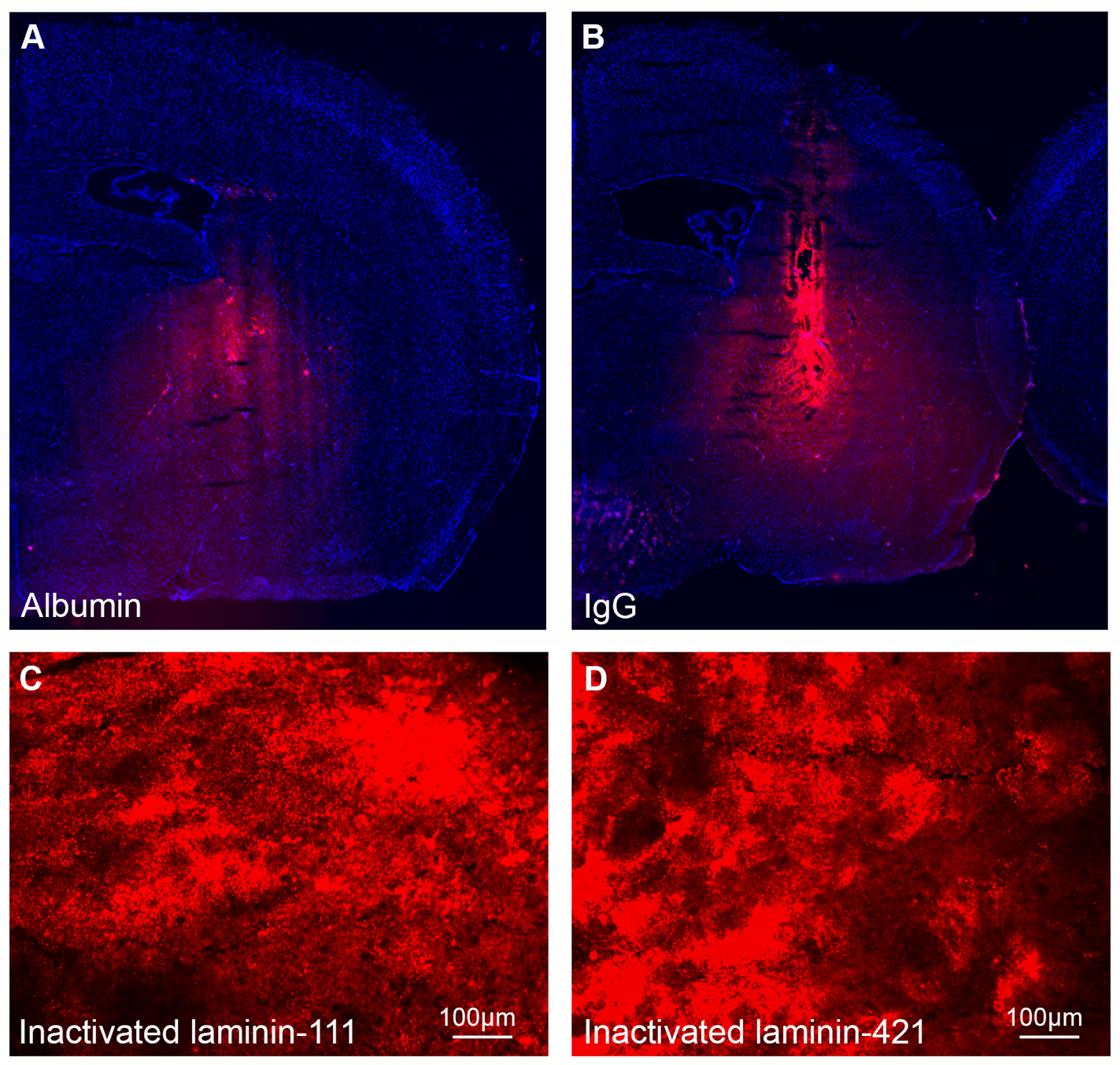
**

**Fig. S1** Albumin, IgG, and heat-inactivated laminins display a typical diffusion pattern at 24 hours after intracerebral injection. **A** and **B** Alexa 555-conjugated albumin and IgG showed a typical diffusion pattern in the brain at 24 hours after intracerebral injection. **C** and **D** Alexa 555-conjugated laminin-111 and laminin-421 were heat-inactivated for 5 minutes at 95°C. Both heat-inactivated laminin isoforms exhibited a typical diffusion pattern in the brain at 24 hours after intracerebral injection.


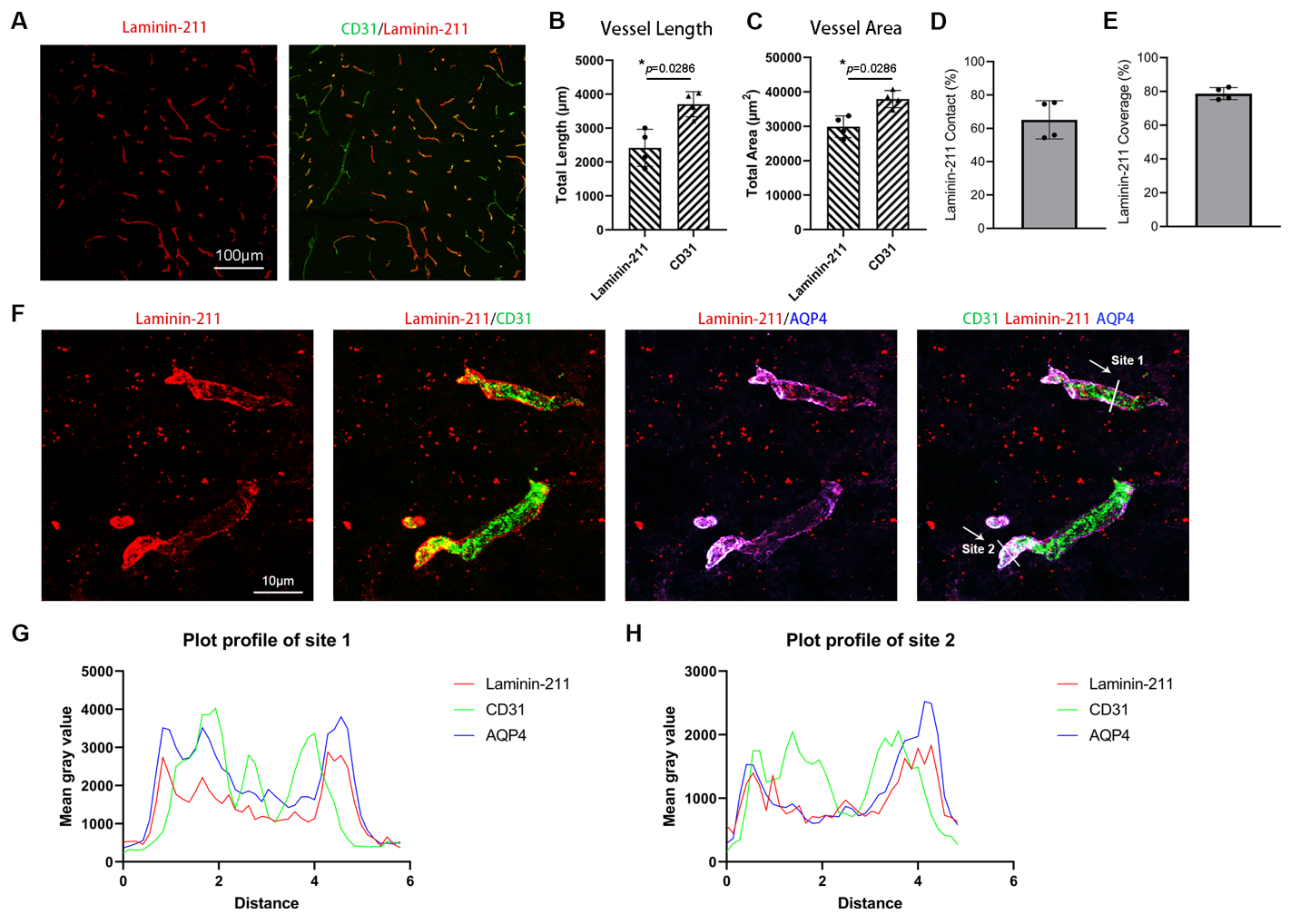


**Fig. S2** Exogenous laminin-211 is enriched in the perivascular space in cerebral vasculature. **A** Representative low-magnification images of Alexa-555 labeled laminin-211 (red) and CD31 (green) in the brain at 24 hours after intracerebral injection. **B** Quantification of vessel length calculated with laminin-211 and CD31 signals. n=4, **p*=0.0286 by Mann-Whitney U test. **C** Quantification of vessel area calculated with laminin-211 and CD31 signals. n=4, **p*=0.0286 by Mann-Whitney U test. **D** Quantification of laminin-211 contact. n=4. **E** Quantification of laminin-211 coverage. n=4. **F** Representative high-magnification images of CD31 (green), Alexa-555 labeled laminin-211 (red), and AQP4 (blue) in the brain at 24 hours after intracerebral injection. White arrows indicated two sites, where spatial profiles of fluorescence intensity were performed. **G**, **H** Spatial profiles of CD31 (green), laminin-211 (red), and AQP4 (blue) along white lines crossing representative capillaries in site 1 (**G**) and site 2 (**H**) in (**F**). Data are represented as mean ± SD.


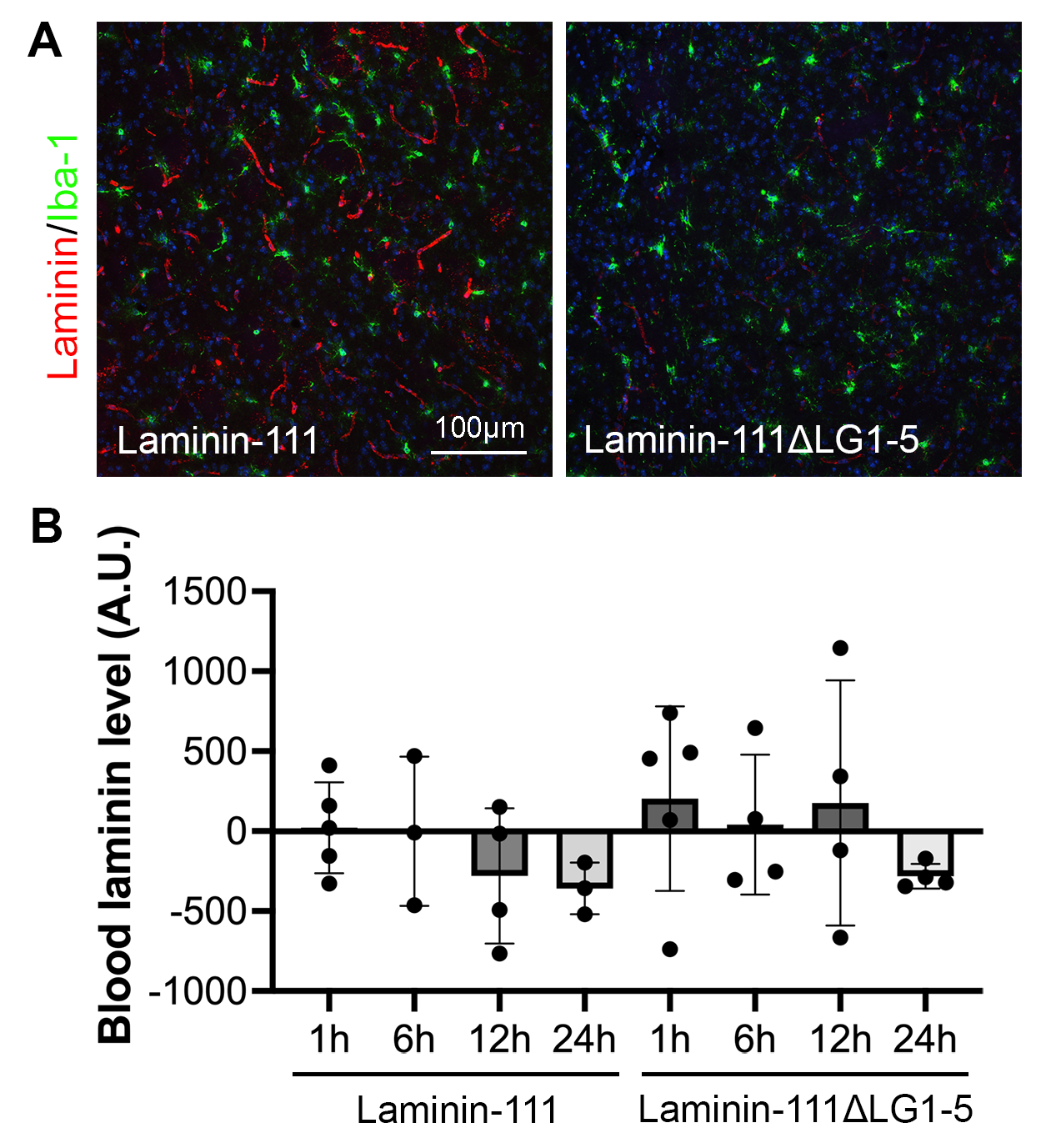


**Fig. S3** Exogenous laminin-111/laminin-111ΔLG1-5 are not eliminated from the brain by microglia/macrophage-mediated phagocytosis or systemic circulation. **A**. Representative images of laminin-111/laminin-111ΔLG1-5 and Iba-1 staining in the brain at 24 hours after intracerebral injection. B. Laminin-111 and laminin-111ΔLG1-5 levels in the blood at 1, 6, 12, and 24 hours after intracerebral injection. n=3-5.


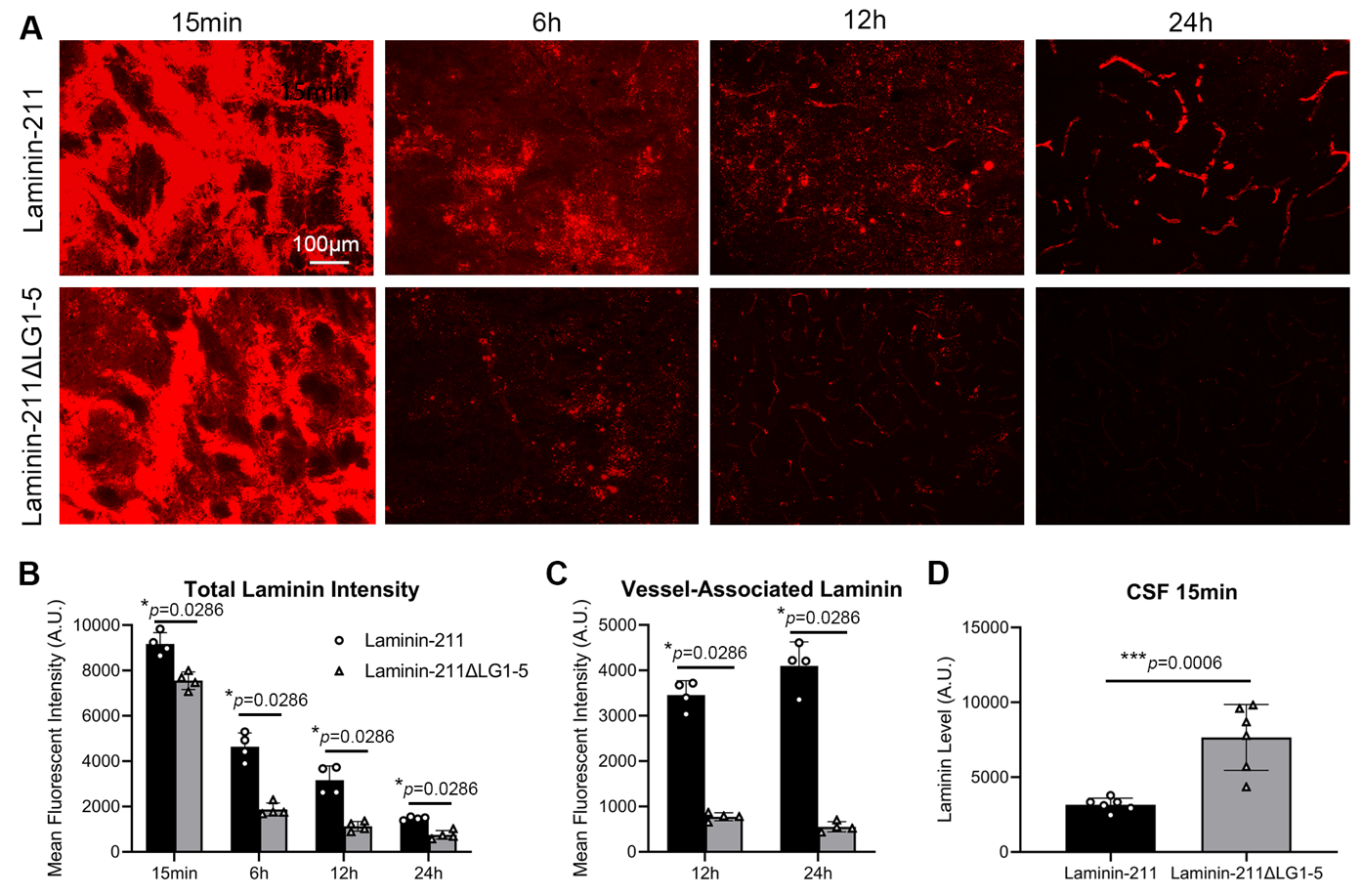


**Fig. S4** Exogenous laminin-211/laminin-211ΔLG1-5 are eliminated from the brain via the perivascular system. **A** Representative images of Alexa-555 labeled laminin-211 (red) and laminin-211ΔLG1-5 (red) in the brain at various time points after intracerebral injection. **B** Quantification of total laminin levels at each time point in (**A**). n=4, **p*=0.0286 by Mann-Whitney U test. **C** Quantification of vessel-associated laminin levels at 12 and 24 hours after intracerebral injection in (**A**). n=4, **p*=0.0286 by Mann-Whitney U test. **D** Quantification of Alexa-555 fluorescent intensity in the CSF at 15 minutes after intracerebral injection. n=6, ****p*=0.0006 by Mann-Whitney U test. Data are represented as mean ± SD.
